# Periodontal Disease and Its Systemic Associated Diseases

**DOI:** 10.1155/2015/153074

**Published:** 2015-08-04

**Authors:** Javier Fernandez-Solari, Paula Barrionuevo, Claudio A. Mastronardi

**Affiliations:** ^1^Department of Physiology, Faculty of Dentistry, University of Buenos Aires, Marcelo T. de Alvear 2142, C1122 AAH Buenos Aires, Argentina; ^2^National Council for Scientific and Technical Research (CONICET), Rivadavia 1917, C1033 AAJ Buenos Aires, Argentina; ^3^Experimental Medical Institute, Medical National Academy, CONICET, Pacheco de Melo 3081, C1425 AUM Buenos Aires, Argentina; ^4^Genome and Predictive Medicine Group, Genome Science Department, The John Curtin School of Medical Research, The Australian National University, Building 131, Garran Road, Canberra, Australia

Inflammatory processes can underlie the etiology of several pathological conditions ranging from metabolic to infectious diseases. Periodontitis, a chronic oral infectious disease, appears to occur as a result of a dysregulated host immune response elicited by subgingival microorganisms occurring in the dental biofilm. Whereas the activity of periodontal pathogens is required, their presence is not sufficient to account for the initiation and progression of periodontal disease. Thus, the combination of the bacterial-elicited insults and the poorly regulated host immune response causes deleterious effects on dentition supporting structures including the periodontal ligament, alveolar bone, and gingival tissues. Furthermore, emerging evidence suggests that periodontal disease can impact on host susceptibility for acquiring other diseases.

The relationship between periodontitis and other pathological conditions could be established by the immunogenic potential of host and/or bacterial products that reach the bloodstream and target distant organs and systems. For instance, lipopolysaccharide, a key component of the outer membrane of Gram-negative bacteria, stimulates host cells to produce a number of potent proinflammatory cytokines such as tumor necrosis factor alpha, interleukin-1, and interleukin-6. It also stimulates the production of other inflammatory mediators, such as prostaglandin E2 and nitric oxide. This inflammatory cascade promotes metalloproteinase matrix release from host tissues and causes deleterious effects in the extracellular matrix and alveolar bone. Thus, periodontitis could start as a local infection, but the triggering of a chronic inflammatory cascade could cause that oral bacteria, LPS, and/or several potent bacterial-induced proinflammatory molecules enter the bloodstream to increase the susceptibility of acquiring other infectious diseases and/or severe pathological conditions such as cardiovascular disease, cerebrovascular diseases, peripheral arterial disease, respiratory diseases, mental disorders (e.g., depression), diabetes, obesity, rheumatoid arthritis, osteoporosis, and complications of pregnancy. Additionally, chronic environmental insults such as exposure to stress, cafeteria diet, smoking, alcoholism, and drugs of abuse could also predispose the host to acquire and/or exacerbate the deleterious effects of periodontal disease and other related conditions. Some of these insults may in turn exacerbate the severity and incidence of periodontal disease by increasing the susceptibility of an impaired host immune response to oral bacteria and their by-products. Indeed, emerging evidence supports the fact that a bidirectional relationship between periodontitis and systemic diseases exists.

Since the understanding of the possible relationship between periodontitis and other systemic diseases still remains to be understood, we decided to edit this special issue on “Periodontal Disease and Its Systemic Associated Diseases” to bring attention on the deleterious impact that this chronic condition could impose on the host. The contributions to this special issue reported on some pathogenic and diagnostic aspects of periodontal disease, and its putative systemic associated conditions.

In the paper entitled “Inflammatory Mediators of Leprosy Reactional Episodes and Dental Infections: A Systematic Review” by D. Cortela et al., the authors conducted a systematic review of primary literature published between 1996 and 2013, to determine the main shared inflammatory mediators in the immunopathological process of dental infections and leprosy reactions, concluding that these biomarkers have predictive and prognostic value to help in the early identification of patients at high risk of periodontal or leprosy diseases.

C-reactive protein (CRP), a well-known marker of inflammation, has been previously reported to predict risk of cardiovascular and cerebrovascular diseases. Interestingly, blood CRP levels also appear to reflect the severity of periodontitis. In the paper “C-Reactive Protein in Peripheral Blood of Patients with Chronic and Aggressive Periodontitis, Gingivitis, and Gingival Recessions” by S. Podzimek et al., the authors compare and evaluate the systemic levels of C-reactive protein (CRP) in peripheral blood samples of patients with chronic and aggressive periodontitis, gingivitis, and gingival recessions and compare them with periodontal clinical parameters, concluding that CRP levels increase subsequently with the severity of the periodontal disease.

The possible bidirectional relationship between periodontitis and systemic diseases was evaluated by R. Nagpal et al. In their paper “The Two-Way Association of Periodontal Infection and Systemic Disorders: An Overview”, the authors revised the literature aiming at explaining a possible bidirectional link between the mechanism of periodontal diseases and systemic/metabolic diseases where both conditions could aggravate each other.

The putative relationship between periodontitis and rheumatoid arthritis was reviewed by V. Araujo et al. In their paper “Relationship between Periodontitis and Rheumatoid Arthritis: Review of Literature”, the authors considered the link between both diseases considering epidemiological aspects, mechanical periodontal treatment, inflammatory mediators, oral microbiota, and antibodies. They concluded that there is a correlation between periodontal disease and rheumatoid arthritis, which could occur as a result of a similar shared imbalance in the immunoinflammatory response.

In the paper entitled “The Influence of Interleukin (IL) 17A and IL17F Polymorphisms on Chronic Periodontitis Disease in Brazilian Patients” by J. Zacarias et al., the authors investigated a possible role of* IL17A* and* IL17F *polymorphisms in the immunopathogenic mechanism of chronic periodontitis in a Southern Brazilian population. Their results demonstrate, through the analysis of genotypes by PCR-RFLP method, that the* IL17A* G197A rs2275913 polymorphism,* IL17A* AA genotype, and A allele were associated with an increased susceptibility to chronic periodontitis but show no evidence for risk or protection associations for* IL17F* T7488C rs763780.

The potential association between type 1 diabetes, a metabolic disease of autoimmune origin, and oral health has been reviewed by M. Novotna et al. In their paper “Periodontal Diseases and Dental Caries in Children with Type 1 Diabetes Mellitus”, the authors considered the literature linking these two conditions. They have thoroughly discussed evidence supporting the fact that type 1 diabetes increases the risk of periodontitis. Moreover, there is compelling evidence showing an association between poorly controlled type 1 diabetes (higher HbA1c levels) and periodontitis. Thus, this review highlights the relevance of controlling oral health in patients undergoing type 1 diabetes.

In the paper entitled “HLA Haplotypes and Genotypes Frequencies in Brazilian Chronic Periodontitis Patients” by E. Â. Sippert et al., the authors studied the associations of HLA with chronic periodontitis (CP) in the Brazilian population to clarify the genetic predisposition to CP. Their results provide evidence that classes I and II HLA polymorphisms are associated with CP. HLA-A*∗*02/B*∗*40 haplotype seems to represent susceptibility factors, and HLA-B*∗*15/DRB1*∗*11 haplotypes were potential protective factors against the disease.

We hope that this special issue will not only be useful in providing insight into new and important aspects related to periodontitis and its systemic associated diseases, but also stimulate the development of novel research ideas and therapeutic strategies in this particular field.

